# Reply to Dal, Z.; Erkan, M. Comment on “Lai et al. Liver Transplantation for the Cure of Neuroendocrine Liver Metastasis: A Systematic Review with Particular Attention to the Risk Factors of Death and Recurrence. *Biomedicines* 2024, *12*, 2419”

**DOI:** 10.3390/biomedicines14092110

**Published:** 2026-09-18

**Authors:** Quirino Lai, Alessandro Coppola, Anna Mrzljak, Maja Cigrovski Berkovic

**Affiliations:** 1 General Surgery and Organ Transplantation Unit, Department of General and Specialty Surgery, Sapienza University of Rome, 00161 Rome, Italy; lai.quirino@libero.it; 2 Department of General Surgery, Sapienza University of Rome, 00161 Rome, Italy; aless.coppola@uniforma1.it; 3 Department of Gastroenterology and Hepatology, Liver Transplant Center, University Hospital Centre Zagreb, 10000 Zagreb, Croatia; anna.mrzljak@gmail.com; 4 Department of Medicine, School of Medicine, University of Zagreb, 10000 Zagreb, Croatia; 5 Faculty of Kinesiology, University of Zagreb, 10000 Zagreb, Croatia

We would like to sincerely thank the Authors for their thoughtful and well-argued commentary on our systematic review addressing liver transplantation (LT) for neuroendocrine neoplasm liver metastases (NEN-LMs) [1]. Their manuscript raises several important points, particularly regarding (i) the presence of cases with an unknown primary tumor, (ii) the role of proliferative activity and Ki-67 thresholds, (iii) the growing relevance of multimodal strategies such as cytoreductive surgery combined with peptide receptor radionuclide therapy (PRRT), and (iv) the ethical and practical implications of living-donor liver transplantation (LDLT) in this setting.

Overall, we believe their commentary is valuable and aligns with the key message of our review: LT for NEN-LM should remain a highly selective strategy, supported by careful patient selection and multidisciplinary decision-making, in a field still largely driven by retrospective evidence.

## 1. Unknown Primary Tumor and the Concept of “Liver-Limited Disease”

The Authors correctly highlight that a non-negligible proportion of patients included in the literature had an unknown or undetected primary tumor, approximating 16% of the pooled reported population [2]. We agree that this represents an important limitation, potentially affecting risk stratification and interpretation of “liver-limited” disease.

However, we would also emphasize that, in clinical practice, the definition of “liver-limited” disease is primarily based on comprehensive staging and functional imaging rather than solely on primary tumor identification. In particular, modern diagnostic pathways (including high-quality cross-sectional imaging and somatostatin receptor-based PET) may exclude extrahepatic disease even when the primary remains occult [3]. Therefore, while the presence of an unknown primary undoubtedly introduces uncertainty, it does not automatically equate to systemic dissemination.

Importantly, the retrospective nature of the available series and inconsistent reporting prevented a meaningful subgroup analysis in our review. We fully agree that future registries and prospective cohorts should specifically report outcomes for patients with occult primaries, since this subgroup may represent a distinct biological and prognostic category.

## 2. Ki-67 Threshold: Selection Cornerstone, but Not a Rigid Dogma

We also agree with the Authors that proliferative activity is one of the strongest determinants of recurrence and survival after LT for NEN-LM. Indeed, in our review, Ki-67 and tumor grade emerged among the most consistently reported prognostic factors.

Nevertheless, we would caution against interpreting the Ki-67 ≤ 2% threshold as an absolute biological boundary. While the Milan criteria historically adopted this cut-off, the available transplant literature does not provide robust comparative evidence supporting Ki-67 < 2% as the only acceptable threshold [4]. As we discussed in our review, only one study had sufficient numerosity to evaluate grade differences in multivariable analysis, while most series remain underpowered for refined stratification [5].

Moreover, Ki-67 is affected by sampling variability, interobserver variability, and intratumoral heterogeneity. These issues are particularly relevant in metastatic disease [6].

Accordingly, when feasible, repeat biopsy or assessment of more than one metastatic lesion could be considered in selected cases to better capture tumor heterogeneity before transplant evaluation, particularly when pathological findings are discordant with the overall clinical or radiological picture [7].

For these reasons, we believe the most reasonable position is that Ki-67 should remain a central selection variable, but future research should aim to determine whether carefully selected patients with slightly higher Ki-67 values (e.g., low G2) may still achieve acceptable long-term outcomes under strict clinical and radiological stability criteria.

## 3. PRRT and Cytoreductive Surgery: Not Alternatives, but Part of Sequencing

We appreciate the Authors’ discussion of cytoreductive surgery and PRRT, and we agree that these modalities deserve increasing attention in the modern therapeutic algorithm for metastatic NENs. The evidence supporting PRRT, including its role in advanced disease and its potential neoadjuvant applications, has expanded substantially, and transplant teams should incorporate these strategies into multidisciplinary planning [8].

At the same time, we would highlight that the emergence of effective systemic and locoregional treatments does not necessarily reduce the relevance of LT. Rather, it reinforces the concept that LT should be viewed as the final step of a carefully planned sequence, ideally reserved for patients who demonstrate favorable tumor biology over time. In this regard, response to PRRT or successful downstaging strategies may become increasingly important tools to refine transplant candidacy, similarly to what has occurred in hepatocellular carcinoma [9].

Therefore, we fully agree with the Authors that multimodal therapy deserves stronger emphasis. However, rather than comparing LT with non-transplant strategies as mutually exclusive options, future studies should investigate how different treatments can be optimally integrated within a sequential therapeutic pathway. In particular, defining the optimal timing of LT after PRRT, systemic therapy or cytoreductive surgery; identifying the duration of disease stability required before listing; and determining which treatment responses should be incorporated into transplant selection criteria are likely to represent the next major challenges in this field.

## 4. Living-Donor Liver Transplantation: Ethical Implications and Risk of Overuse

We also acknowledge the Authors’ concern regarding the potential overuse of LT for NEN-LM in LDLT settings, given that donor risk can only be ethically justified when recipient benefit is robust and well-established.

We agree that LDLT introduces additional ethical complexity and should be addressed explicitly in future transplant oncology research [10]. In our systematic review, LDLT-specific evidence was scarce and inconsistently reported, preventing meaningful stratified analyses. This is a key limitation of the available literature, and we concur that LDLT should not be promoted broadly for NEN-LM outside strict selection frameworks and preferably within prospective registries.

At the same time, we would underline that the risk of inappropriate expansion is not unique to LDLT [11]. Even in deceased-donor transplantation, access to LT is already constrained by national allocation policies and multidisciplinary selection committees, which must balance transplant oncology indications against patients with end-stage liver disease or acute liver failure. Consequently, candidates with NEN-LM are often assigned lower priority, reflecting not only the ongoing uncertainty regarding long-term benefit but also the ethical imperative to allocate a scarce public resource where the expected benefit is greatest.

For this reason, the development of robust evidence defining which patients derive the greatest survival benefit from LT is essential. More precise selection criteria would not only reduce the risk of overuse, particularly in LDLT, but also facilitate a more equitable integration of NEN-LM into deceased-donor allocation systems. However, in the absence of robust prospective data, LDLT for NEN-LM should be restricted to well-designed research protocols or registries.

## 5. Conclusions

In conclusion, we thank the Authors for their insightful commentary, which reinforces several key limitations and future directions identified in our review. We agree that (i) unknown primary tumors represent an important confounder, (ii) Ki-67 remains a cornerstone variable but should be interpreted with biological nuance and methodological caution, (iii) PRRT and cytoreductive strategies are increasingly relevant in patient selection and sequencing, and (iv) LDLT introduces additional ethical concerns that should be explicitly incorporated into future research and policy discussions.

We hope that ongoing international collaborations and prospective registries will help clarify these unresolved issues and ultimately define the most appropriate role of LT within the evolving multidisciplinary management of NEN-LM.

## Data Availability

No new data were created or analyzed in this study. Data sharing is not applicable to this article.

## Abbreviations

The following abbreviations are used in this manuscript:

LT	Liver transplantation
NEN-LM	Neuroendocrine neoplasm liver metastasis
LDLT	Living-donor liver transplantation
PRRT	Peptide receptor radionuclide therapy
